# Preamble Design and Noncoherent ToA Estimation for Pulse-Based Wireless Networks-on-Chip Communications in the Terahertz Band

**DOI:** 10.3390/mi16010070

**Published:** 2025-01-08

**Authors:** Pankaj Singh, Sung-Yoon Jung

**Affiliations:** Department of Electronic Engineering, Yeungnam University, Gyeongsan 38541, Republic of Korea; pankaj_singh86@ynu.ac.kr

**Keywords:** THz channel modeling, chip multiprocessor, graphene, iWISE architecture, intra-chip wireless interconnect, noncoherent detection, synchronization, THz nanocommunications, time-of-arrival (ToA), wireless networks-on-chip (WiNoC)

## Abstract

The growing demand for high-speed data transfer and ultralow latency in wireless networks-on-chips (WiNoC) has spurred exploration into innovative communication paradigms. Recent advancements highlight the potential of the terahertz (THz) band, a largely untapped frequency range, for enabling ultrafast tera-bit-per-second links in chip multiprocessors. However, the ultrashort duration of THz pulses, often in the femtosecond range, makes synchronization a critical challenge, as even minor timing errors can cause significant data loss. This study introduces a preamble-aided noncoherent synchronization scheme for time-of-arrival (ToA) estimation in pulse-based WiNoC communication operating in the THz band (0.02–0.8 THz). The scheme transmits the preamble, a known sequence of THz pulses, at the beginning of each symbol, allowing the energy-detection receiver to collect and analyze the energy of the preamble across multiple integrators. The integrator with maximum energy output is then used to estimate the symbol’s ToA. A preamble design based on maximum pulse energy constraints is also presented. Performance evaluations demonstrate a synchronization probability exceeding 0.98 for distances under 10 mm at a signal-to-noise ratio of 20 dB, with a normalized mean squared error below $10^{-2}$. This scheme enhances synchronization reliability, supporting energy-efficient, high-performance WiNoCs for future multicore systems.

## 1. Introduction

The evolution of chip multiprocessors (CMPs) and the adoption of the network-on-chip (NoC) paradigm [1,2] mark significant milestones in integrated circuit development. In CMP environments, traditional bus-based on-chip interconnects have been replaced by more efficient and robust NoCs. Initially, NoCs relied on wired solutions, which presented challenges related to delay, power consumption, and chip area, particularly as more cores were integrated [3,4,5]. In modern computing, multicore architectures dominate, where a single chip houses numerous independent processor cores and a dedicated portion of on-chip memory. These processors operate concurrently, utilizing shared memory for data exchange. To enhance computing performance, the trend is to integrate more cores onto a single chip [6], which exacerbates the performance issues of wired NoCs. As a result, alternative interconnect technologies have been proposed [5,7], including wireless communication within the chip [8,9], known as wireless networks-on-chips (WiNoC). Advances in shrinking transceivers and antennas have opened new opportunities for WiNoC development [8,9,10,11], enabling chip-scale communication that enhances the capabilities of multicore and multichip systems.

Wireless communication within on-chip networks offers several advantages, including reduced propagation delay, adaptability, and improved scalability in terms of latency, data transfer rates, and energy efficiency [12,13]. Currently, WiNoC complements traditional wired NoC. However, existing WiNoCs still require relatively large metallic antennas to enable wireless communication in the millimeter-wave (mmWave) frequency band. To address the issue, pioneering work [14] proposed the use of nanoscale WiNoCs featuring graphene nanoantennas, known as graphene-enabled wireless networks-on-chips (GWiNoC). This approach utilizes the graphene-based nanoantennas that operate in the terahertz (THz) band, facilitating wireless communication between multiprocessor cores [15,16,17]. These nanoantennas are exceptionally compact, measuring only a few micrometers–two orders of magnitude smaller than metallic antennas [18,19]. Moreover, they exhibit inherent tunability [20,21,22,23]. In addition to antennas, significant progress has been made in developing small on-chip THz nano-generators [24], detectors [25], and demodulators [26] for THz-WiNoC systems.

Motivated by the potential of THz-WiNoC, several THz channel models have been proposed [27,28,29,30,31,32]. Reference [28] uses electromagnetic (EM) field distributions within WiNoC stratified media, employing the Sommerfeld integration method and verifying results with a full-wave solver. Based on these theoretical results, the authors characterize the WiNoC channel in terms of path loss, showing a high frequency dependency caused by surface and guided waves. The work in [29] investigates physical propagation modeling within dielectric mediums, considering the propagation electric field, absorption, phase, reflection, and transmission coefficients. Subsequently, a ray-tracing-based WiNoC channel is introduced and validated with measurement data. Reference [30] provides a comprehensive survey of existing channel modeling efforts, categorizing them into mmWave, THz band, and optical wireless models. In addition to channel models, studies [28,33,34] explore the feasibility of on-off keying modulation for WiNoC communications.

Beyond channel models and modulation schemes, synchronization is crucial for the success of any communication system, ensuring orderly and coherent data transmission, reception, and processing. In conventional wired on-chip networks, synchronization is relatively straightforward owing to well-defined signal propagation characteristics. However, as chip technologies scale and operating speeds increase, achieving global synchronization becomes more challenging [2]. Approaches like globally asynchronous, locally synchronous systems [35] introduce local synchronization islands to address these challenges, allowing integration of intellectual property cores with differing timing characteristics. Techniques such as phase- and delay-locked loops (PLLs and DLLs, respectively) are also increasingly used for intra-chip synchronization, though they consume additional power [2]. The wireless nature of WiNoC presents unique challenges that require innovative solutions. Although energy supply is typically sustained in computer systems, THz-WiNoC demands low power consumption [6] to limit heat dissipation on the chip. Due to the ultrashort width of THz pulses, even minor timing errors can result in missed pulses, making perfect synchronization crucial. Moreover, extreme attenuation and dispersion in the THz band can distort signals, complicating time-of-arrival (ToA) estimation. In contrast, at lower frequencies like mmWave, pulses are longer, and timing requirements are less stringent. Consequently, synchronization schemes at these frequencies can rely on less precise techniques, such as PLLs or pilot signals, which are unsuitable for THz frequencies due to the very short duration of THz pulses (on the order of femtoseconds). While UWB systems also deal with short pulses, they operate at lower frequencies with different propagation characteristics. Although noncoherent detection and energy-based synchronization schemes have been explored in UWB systems [36,37,38], the high attenuation and dispersion in the THz band introduce challenges not found in UWB communications. Several synchronization schemes for THz communication have been proposed [39,40], but none specifically address the WiNoC scenarios.

In this study, we propose a preamble-aided synchronization scheme for ToA estimation in pulse-based WiNoC in the THz band (0.02 to 0.8 THz). The main contributions of this study are as follows.

- We design a preamble consisting of a signaling part and a guard interval to achieve synchronization. The design is based on solving a nonlinear optimization problem to optimize the energy allocated to the pulses.
- We consider a THz-WiNoC channel based on the iWISE-256 architecture, a popular WiNoC framework [8]. The package uses flip-chip technology, and the chip design is modeled as a multilayer structure. Specifically, we adopt the EM field analytical channel model in [28] to derive the channel frequency and impulse responses. Additionally, we present the noncoherent energy-detection receiver architecture, which is simple and energy-efficient.
- Based on the energy-detection receiver, we propose a synchronization scheme for ToA estimation. We evaluate the performance of this scheme in terms of synchronization probability and error, providing a comprehensive assessment of its effectiveness.

To the best of our knowledge, no prior work has addressed the synchronization issue in the THz-WiNoC communications.

The remainder of this paper is organized as follows. Section 2 discusses the preamble signal structure and the current state-of-the-art in THz signal generation and reception. Section 3 presents the iWISE-256 WiNoC architecture and derives the channel model in terms of frequency and impulse responses. Section 4 introduces the synchronization scheme based on the noncoherent energy-detection receiver, providing a detailed mathematical model and optimization of the preamble signal energy. Section 5 presents simulation results based on various system design parameters, including the effects of channel, number of integrators, and preamble repetitions on synchronization probability, as well as average mean squared error. Finally, Section 6 concludes the study.

## 2. Preamble Definition

**Figure 1:**
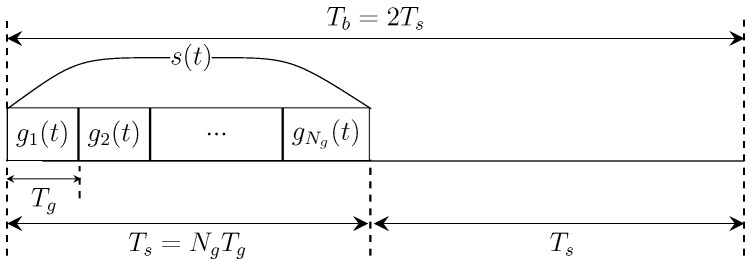
Structure of the preamble signal [40].

The preamble signal structure is depicted in Figure 1. The preamble consists of two parts: a signaling part, s(t), and a guard interval, both of which have equal duration $T_{s}$. As a result, the total duration of the preamble is $T_{b}=2T_{s}$. The signaling part comprises $N_{g}$ pulses, each with a width of $T_{g}$. This structure ensures reliable synchronization by separating consecutive pulses and allowing sufficient time for energy collection during the preamble period. In a network, to ensure robust synchronization, it is necessary to transmit the preamble signal multiple times. Assuming that the preamble signal is transmitted *N* times for reliable synchronization, hence, the set of preamble signals is given by

(1) $$x\left(t\right)=\sum_{n=0}^{N-1}\left(\sum_{i=1}^{N_{g}}g_{i}\left(t-iT_{g}-nT_{b}\right)\right),$$

where $g_{i}\left(t\right)$ represents the ultrashort THz pulse of width $T_{g}$. The total preamble energy is given by $E_{s}=\sum_{i=1}^{N_{g}}E_{g_{i}}$, where $E_{g_{i}}$ represents the energy of the *i*th pulse.

To meet the requirements of THz-WiNoC systems, we propose the use of a femtosecond-long Gaussian pulse, defined by the equation

(2) $$g\left(t\right)=\frac{\alpha }{\sqrt{2\pi }\sigma }e^{-{\left(t-\mu \right)}^{2}/\left(2\sigma^{2}\right)},$$

where α denotes the normalization constant, σ represents the standard deviation of the pulse, which controls the width, and μ indicates the time delay. The power spectral density of these pulses predominantly features frequency components in the THz band [41].

### THz Signal Generation and Detection

Conventional electronic transmitters and receivers used for radio and microwave frequencies are compact, affordable, and operate efficiently at room temperature. However, developing similar devices for the THz band presents significant challenges due to the limited availability of suitable hardware, particularly compact and cost-effective THz sources [42,43,44]. At present, THz waves can be generated by either up-converting the frequency of electronic sources to the desired THz range or down-converting signals from optical sources. For WiNoC communications, THz-band signal generators must be compact, fast, energy-efficient, and tunable. Traditionally, monolithic integrated circuits made from materials such as silicon, silicon germanium, indium phosphide, and gallium nitride have demonstrated the capability to operate at frequencies ranging from 0.1 to 1 THz [45]. For higher frequencies, photonic devices, especially quantum cascade lasers (QCLs), are used. QCLs operate through laser mixer or femtosecond laser pulses, capable of exceeding a few THz in frequency and producing average powers up to several milliwatts. A novel approach involves intracavity difference-frequency generation in dual-wavelength mid-infrared QCLs, which can operate at room temperature and generate up to 1.9 mW of power within the 1–6 THz range [42]. Resonant tunnel diode oscillators can operate at higher THz frequencies but typically produce relatively low power levels (<1 mW).

There has also been progress toward developing more compact signal generators that use single high-electron-mobility transistor (HEMT) based on III-V compound semiconductors like gallium nitride and graphene [44,46]. Research has shown that surface plasmon-polariton (SPP) waves can be excited in the channel of a HEMT with a nanometric gate length, either through electrical or optical pumping. When a voltage is applied across the drain and source of the HEMT, electrons move rapidly, inducing SPP waves due to the material’s energy band structure. However, at room temperature, these waves are heavily damped, resulting in very short, broadband, incoherent SPP waves that last only tens of femtoseconds. Despite this limitation, innovations like graphene-based nanoantennas and nanotransceivers can improve the performance of WiNoC by enabling tunable and efficient transmission characteristics. It is important to note that while WiNoC environments typically have sufficient power, the actual power levels of the transmitted preamble or data signal must still be kept low (∼μW) to minimize heating, avoid interference with other components, maintain low operational temperatures, and extend the battery life in portable devices [6].

In terms of THz detection, graphene and its derivatives, such as carbon nanotubes (CNTs), play a central role [47,48]. Graphene, also known as a Dirac material, has electron and phonon-free paths much longer than those in conventional materials and a remarkably high electron mobility (up to 200,000 cm^2^/Vs), making it ideal for devices like high-frequency ballistic transistors capable of operating in the THz range. Additionally, graphene and CNTs facilitate plasma wave oscillations due to their quasi-ballistic electron transport, allowing THz radiation to excite these waves between the source and drain of a field-effect transistor (FET), producing a DC output. The sensitivity of these detectors can be tuned via the gate bias, taking advantage of graphene’s high electron mobility and the resonant cavity properties of aligned CNTs. Graphene FETs, coupled with antennas, have demonstrated significant progress in THz detection by leveraging the material’s ability to control carrier concentration and enhance plasmon propagation [47]. In addition to plasma waves, the hot carrier-assisted photoconduction technique also enables THz wave detection [49,50]. This technique involves the excitation of electrons by THz radiation, which results in increased electron movement within the graphene, generating an electrical signal. The proposed device can efficiently convert THz radiation into an electrical signal at room temperature and does so rapidly (within approximately one microsecond) [43].

## 3. WiNoC Architecture and Channel Model

WiNoC channel models in the mmWave band, utilizing UWB radio interconnects, have been studied for over two decades [31,51,52]. However, WiNoC channel models for the THz band were first proposed over ten years ago [8,9]. In [8,9], a simple two-ray model for intra-chip channels was proposed. This model considers one line-of-sight (LoS) path and a reflected path from the plane on which the transmit and receiver antennas are located. Path loss is estimated using a log-distance formula adapted from conventional terrestrial models. The study found that path loss is linear with transmission distance for frequencies up to 10 THz and antenna heights of 10 µm. However, these models did not account for the chip packaging. Interest in GWiNoC grew simultaneously [14,15], leading to the proposal of a channel model for GWiNoC environments [32]. This model, based on reflection, encompasses path loss, dielectric propagation loss, and molecular absorption attenuation in the frequency range of 1–1.6 THz. However, it only considers a simple two-ray channel in a basic chip package structure. Moreover, it assumes an open-chip package with several gases inside, neglecting the intra-chip material properties. The impact of graphene nanoantennas on wave propagation and path loss remains unclear. In [27], THz propagation in CPU architecture is discussed, and a tool based on ray-tracing methodologies is proposed. This tool uses surface tessellation to divide the structure into miniature segments. However, no mathematical models are presented.

For a more comprehensive WiNoC channel design, modeling the chip architecture is necessary. A WiNoC chip design based on the iWISE-256 structure, a popular WiNoC framework, was introduced in [8]. The iWISE-256 architecture includes 256 cores for CMPs, organized into four iWISE-64 sub-architectures, each comprising 64 cores. Cores are grouped in clusters, sets, and groups, with the final iWise-256 architecture formed by four adjacent groups. The chip package employs flip-chip technology and incorporates a heat sink. The cross-section of the WiNoC structure was later adopted in [29]. Using the ray-tracing technique, a channel model was developed, and THz-band path gain was computed in the 0.02–1 THz range, showing fluctuations due to propagation. Unlike free-space THz channels, atmospheric absorption is negligible in WiNoC, as the propagation medium consists of dielectric materials within a sealed chip package. More recently, a THz-WiNoC channel model using EM field analysis was developed [28,53]. The channel is characterized by path loss in the 0.02–0.8 THz range, exhibiting oscillating and periodic behaviors due to surface and guided wave propagation. Path loss peaks and dips correspond to the frequency differences between adjacent wave modes. The authors summarized their work in [30]. Additionally, ref. [34] considers a simple log-distance path loss model for the 0.22–0.32 THz band.

In this study, we adopt the channel model presented in [28,53] due to its mathematical analysis of the WiNoC channel in the 0.02–0.8 THz range. This model is based on a 40 mm × 40 mm iWISE-256 architecture with a multilayer chip structure. Unlike other studies, this model considers the impact of the chip package and heat spreader, which were neglected in previous works. The model, developed using EM field analysis, is validated using a high-frequency structure simulator (HFSS), a full-wave EM solver. The THz-WiNoC channel is derived by modeling unpackaged chips embedded on the package substrate and in contact with a heat sink. In flip-chip packaging, the chip is inverted and attached to the package substrate, with the chip’s surface facing the substrate [30]. Interconnections between the chip and substrate are made using solder bumps, with underfill material enhancing stability.

**Figure 2:**
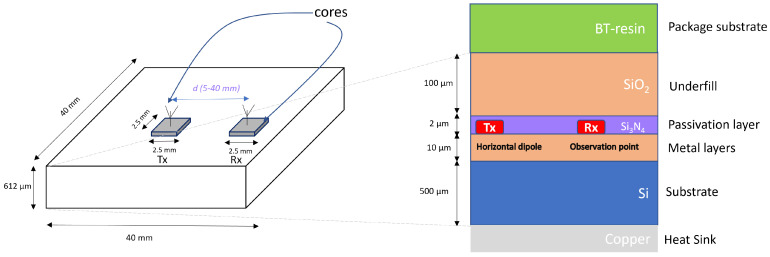
Cross-section of the WiNoC structure [28,32].

Figure 2 shows the cross-section of an un-flipped chip [28,53]. The top layer consists of the package substrate, featuring Bismaleimide Triazine (BT)-resin as the dielectric material. A 100 µm thick silicon dioxide, SiO_2_, underfill layer is used, with a 2 µm thick passivation layer of silicon nitride, Si_3_N_4_, containing integrated on-chip antennas. The metal layers total 10 µm in thickness and are filled with SiO_2_. The silicon substrate is 500 µm thick, with copper beneath it for heat dissipation. Given that the core dimensions are 2.5 mm in length and width, the minimum spacing between adjacent transmitters and receivers is 5 mm. With the total iWise-256 dimensions being 40 mm × 40 mm × 612 µm, the wireless transmission distance ranges from 5 to 40 mm.

Path loss can be computed theoretically by analyzing the EM fields in the six-layer WiNoC structure [28,53]. A Hertizian dipole is considered in a grounded stratified medium of infinite dimension. Path loss, defined as the ratio of transmitted power, $P_{t}$, to received power, $P_{r}$, is calculated using complex Poynting theorem [28]

(3) $$\begin{array}{cc} A(f) & =\frac{P_{t}}{P_{r}}=\frac{P_{t}}{A_{\mathrm{eff}}\left|\langle S\left(\rho ,z,f\right)\rangle \right|}, \end{array}$$

where $A_{\mathrm{eff}}$ is the effective receiving area of the antenna, and 〈S(ρ,z,f)〉 represents the time-averaged Poynting vector, indicating the power density at the observation point (ρ,z) at frequency *f* [28]. The complex Poynting power density is defined as the cross-product of the electric field and the complex conjugate of the magnetic field. By inserting the values of $A_{\mathrm{eff}}$, $P_{t}$, and the Poynting vector, the final path loss is given by [28]

(4) A(f)=8πf4μ05/2ϵ03/2I2L23Re2{ExHy*}+Re2{ExHz*},

where *f* denotes the wave frequency, $\mu_{0}$ and $\epsilon_{0}$ indicate the permeability and permittivity of the 0th layer (i.e., the passivation layer), respectively, *I* is the current intensity, *L* is the dipole length, and $E_{x}$, $H_{y}$, and $H_{z}$ are *x*-, *y*-, and *z*-direction components of the electric, *E*, and magnetic fields, *H*, respectively. Considering the above path loss in the THz band, the THz-WiNoC channel frequency response can be defined as

(5) $$H\left(f\right)=\frac{1}{\sqrt{A(f)}}\exp\left(-j2\pi f\tau \right),$$

where τ indicates the time delay. The channel impulse response, h(t), is obtained through the inverse Fourier transform:

(6) $$h\left(t\right)=F^{-1}\left\{H\left(f\right)\right\}.$$

Since this in verse Fourier transform does not have a closed-form solution, it will be numerically computed in our simulations.

**Figure 3:**
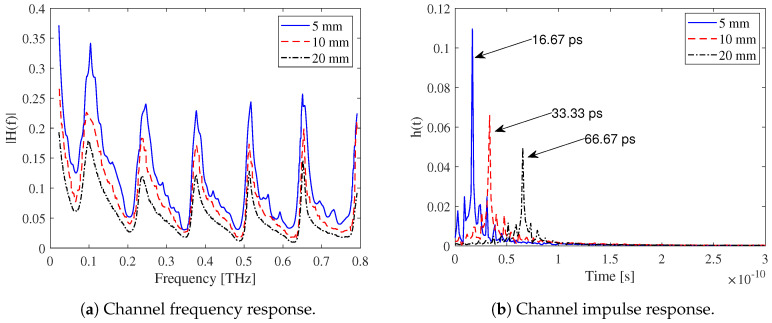
WiNoC ch annel model for the horizontal dipole in the stratified medium [28,53].

Figure 3 shows the channel response for a CMOS-based WiNoC channel in the THz range for three different path lengths: 5, 10, and 20 mm. As the distance increases, heavy attenuation is observed in both the frequency and impulse response profiles. In Figure 3a, the oscillating and periodic behavior of the frequency response is evident due to the interaction of surface and guided waves [28]. The WiNoC channel forms a coated dielectric waveguide, supporting both surface and guided waves. The metal layers act as reflective boundaries, while the silicon substrate serves as the guiding medium. The heat sink influences the overall wave propagation environment (cf., Figure 2). Constructive interference at specific frequencies enhances the field intensity, leading to peaks in the frequency response, while destructive interference causes dips. These periodic behaviors correspond to the difference in cutoff frequencies between adjacent surface wave and guided wave modes [28]. As the transmitter–receiver distance increases, the frequency response gain decreases. In Figure 3b, the THz-WiNoC channel impulse response is shown, with delay observed in addition to attenuation. The impulse response peaks correspond to the delay at the receiver, matching the respective distances from the source. Furthermore, the temporal spread of the impulse response indicates dispersion in the WiNoC channel, resulting in signal spreading over time.

### THz Channel Influence on the Synchronization Scheme Design

This section discusses how the characteristics of the THz channel influence the design of the proposed synchronization scheme:

- Attenuation effects: The THz frequency band experiences significantly higher attenuation compared to lower-frequency bands due to molecular absorption and scattering. In the context of intra-chip WiNoC, while atmospheric absorption is negligible, material properties such as silicon substrate and packaging layers contribute to signal loss. To address this, the synchronization scheme employs an energy-detection-based receiver, which accumulates energy over time rather than relying on the precise phase or polarity of the received signal. This design mitigates the impact of attenuation, ensuring accurate synchronization.
- Dispersion effects: In the WiNoC environment, the stratified medium causes dispersion as signals reflect and interact with various chip layers. To manage these effects, the scheme incorporates multiple integration windows, with each integrator accumulating energy over a specific time interval. The peak energy output among these windows is then used to estimate the ToA. This design ensures robust synchronization despite dispersion induced by the THz channel.
- Ultrashort pulse durations: The ultrashort duration of THz pulses, typically on the order of femtoseconds, demands precise timing to avoid missing pulses. Even minor timing offsets can lead to significant synchronization errors. To address this, the preamble design includes a guard interval between consecutive symbols, reducing the likelihood of inter-symbol interference. Additionally, the energy collection mechanism employs high-resolution integration windows to efficiently capture the energy of these ultrashort pulses.
- Time-varying channel effects: Although the intra-chip channel is relatively stable, factors such as thermal variations or fabrication inconsistencies can introduce time-varying effects. To enhance reliability under such conditions, the preamble signal is transmitted multiple times (*N* repetitions). This repetition averages out temporal variations and increases robustness against minor channel fluctuations.
- Energy efficiency and hardware simplicity: Energy efficiency and minimal hardware simplicity are essential in WiNoC systems to reduce power consumption and heat dissipation. To meet these constraints, the synchronization scheme avoids complex coherent detection mechanisms, such as PLLs. Instead, it uses a simple, noncoherent energy detection approach, which significantly reduces hardware and computational complexity while maintaining synchronization accuracy.
- Channel modeling insights: The synchronization scheme design is informed by an EM-field-based WiNoC channel model that accounts for guided and surface wave effects. This model predicts channel responses with high accuracy and helps optimize parameters such as preamble structure and integration window settings, ensuring compatibility with the specific characteristics of THz-WiNoC systems.

By addressing the unique challenges posed by the THz channel, the proposed synchronization scheme delivers high reliability and accuracy, supporting efficient pulse-based communication in WiNoC systems.

## 4. Proposed Synchronization Mechanism

### 4.1. THz-WiNoC Receiver Architecture

**Figure 4:**
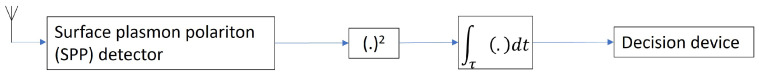
Block diagram of a noncoherent energy-detection-based WiNoC receiver.

To elucidate the synchronization strategy, a noncoherent WiNoC receiver structure is proposed, as illustrated in Figure 4. Noncoherent receivers are particularly suitable for pulsed THz nanosystems due to their simplicity in implementation [39,54]. While WiNoC environments benefit from sustained energy supply, they require efficient signal processing algorithms to minimize resource consumption and conserve device battery power. Consequently, a noncoherent energy-detection receiver is proposed for pulse-based THz-WiNoC communications. The proposed receiver operates in the THz band, leveraging graphene-based plasmonics for signal propagation. The architecture begins with an SPP detector, followed by a noncoherent energy detection scheme. Unlike coherent receivers, which require components such as a template signal generator, PLL, and timing control blocks, the key element of the noncoherent receiver is a squaring device that eliminates the phase or polarity information. The subsequent integrator captures the signal energy over a duration τ, significantly reducing both hardware and computational complexity. The received preamble signal can be expressed as:

(7) r(t)=x(t)∗h(t)+n(t),

where n(t) is Gaussian noise. Given WiNoCs burst communication requirements, the noncoherent receiver ensures rapid signal acquisition and synchronization.

### 4.2. Synchronization Scheme

**Figure 5:**
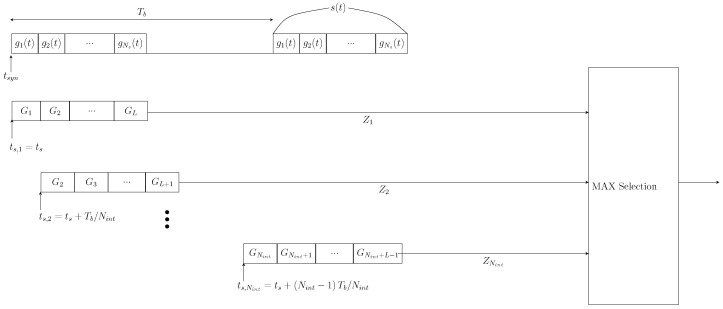
Mathematical representation of the proposed synchronization process and ToA estimation $\left(t_{s}=T_{b}/N_{int}\right)$ [40].

To maintain the receiver’s low-complexity design, the synchronization process is built on energy collection principles [37,40,55,56]. As shown in Figure 5, multiple integrators (or integration windows) accumulate preamble energy across adjacent time intervals. Specifically, $N_{int}$ parallel integrators store the collected energies in a sample-and-hold capacitor network for subsequent processing. A relative comparison of the voltages across $N_{int}$ capacitors is performed, enabling the peak detector to identify the integrator with the highest energy. The decision device identifies the starting point of the selected integrator as the accurate synchronization point.

The proposed synchronization process is depicted in Figure 5. It is assumed that $N_{int}$ integrations are required to collect the entire preamble energy. The observation window of the integrator is divided into sub-windows, each of size $T_{b}/N_{int}$. Compared to the traditional single-window method, the sampling rate increases by a factor of *L*, where *L* is the total number of sub-windows, given by $L=\left(T_{s}/T_{b}\right)N_{int}$. The sub-window width may or may not equal the pulse width, $T_{g}$. Given $T_{b}=2T_{s}$, the total number of sub-windows become $L=N_{int}/2$. In this scenario, the starting point of the γth integrator is defined as [40]

(8) $$t_{s,\gamma }=t_{s,1}+\left(\gamma -1\right)\frac{T_{b}}{N_{int}},$$

where $t_{s,1}$ represents the starting point of the first integrator. The output of the γth integrator is expressed by [40]

(9) $$Z_{\gamma }=\sum_{l=\gamma }^{\gamma +(L-1)}G_{l},\;\gamma =1,...,N_{int},$$

where

(10) $$G_{l}=\int_{t_{s,\gamma }+\left(l-1\right)T_{b}/N_{int}}^{t_{s,\gamma }+lT_{b}/N_{int}}r^{2}\left(t\right)\mathrm{dt}.$$

Here, r(t) denotes the received signal. The estimated ToA of the symbol is given by [40]

(11) $${\hat{t}}_{syn}=t_{s}+\left(\gamma^{*}-1\right)\frac{T_{b}}{N_{int}},$$

where

(12) $$\gamma^{*}=\max_{\gamma }\left(Z_{\gamma }\right).$$

The correct synchronization time, ${\hat{t}}_{syn}$, is defined as the delay that maximizes the preamble signal energy collection. In the AWGN scenario, this corresponds to the beginning of the preamble slot, where all signal energy appears in one integrator. However, in a time-dispersive channel, $N_{int}$ integrators collectively contain the preamble energy. The correct synchronization point is the delay maximizing the energy collection, i.e., the time instant corresponding to the maximum integrator output is considered the nearest achievable synchronization point, determining the estimated ToA of the symbol. The sub-integrator width can be adjusted to delay or overlap, ensuring comprehensive pulse energy collection. The energy collection process always begins at the preamble signal onset. The synchronization time accuracy depends on the number of integrators, $N_{int}$, with ${\hat{t}}_{syn}$ achievable within the error range [40]:

(13) $${\hat{t}}_{syn}\in \left[t_{syn}-\frac{T_{b}}{2N_{int}},t_{syn}+\frac{T_{b}}{2N_{int}}\right],$$

where $t_{syn}$ denotes the exact ToA. The equation above highlights the critical role of sub-window width in determining synchronization accuracy. A higher degree of synchronization time accuracy can be achieved by increasing $N_{int}$, as this reduces the sub-window width (i.e., $T_{b}/N_{int}$). However, a large $N_{int}$ also results in a greater number of outputs to compare, therefore increasing complexity, prolonging processing time, and constraining performance. Conversely, if the duration of the sub-windows exceeds the pulse width, $T_{g}$, maintaining a lower $N_{int}$ can still provide a reasonable synchronization probability. However, this approach sacrifices some synchronization time accuracy due to the expanded error range. Thus, the trade-off between synchronization accuracy, processing complexity, and performance should be carefully considered when evaluating the proposed scheme.

### 4.3. Preamble Structure Design

Designing a preamble signal involves determining how to distribute the preamble energy, $E_{s}$, across each $g_{i}\left(t\right)$ by selecting $G_{l}$ and the corresponding $g_{i}\left(t\right)$ to satisfy $\int_{T_{g}}g_{i}^{2}\left(t\right)dt=E_{g_{i}}$ [57]. The goal is to ensure that each element of $Z\left(={\left[Z_{1},Z_{2},\ldots ,Z_{N_{int}}\right]}^{T}\right)$ closely approximates the ideal synchronization point, $Z^{\mathrm{ideal}}={\left[E_{s},0_{\left(N_{int}-1\right)\times 1}\right]}^{T}$. This occurs when the energy output of the first integrator, starting near the signal’s beginning, reaches its maximum value. For simplicity, we assume that the period of each sub-window is an integer multiple of $T_{g}$, such that $T_{b}/N_{int}=mT_{p}$, and $T_{s}$ is a multiple of slot duration, i.e., $T_{s}/mT_{p}=L$, where *m* and *L* are integers. Additionally, all pulses are constrained to have equal energy. Each pulse in the preamble, $g_{i}\left(t\right)$, where $1\leq i\leq N_{g}$, is defined as [36]

(14) $$g_{i}\left(t\right)=\sqrt{E_{g_{i}}^{\mathrm{opt}}}p\left(t\right),$$

where

(15) $$E_{g_{i}}^{\mathrm{opt}}=\frac{1}{m}G_{\left\lfloor \frac{i}{m}\right\rfloor +1}^{\mathrm{opt}},$$

and p(t) is a basic pulse with unit energy and duration $T_{p}$, satisfying $\int_{T_{p}}p{\left(t\right)}^{2}dt=1$. Here, $E_{g_{i}}^{\mathrm{opt}}$ is the optimal pulse energy allocated to each $g_{i}\left(t\right)$. The $G_{l}^{\mathrm{opt}}$ for 1≤l≤L is obtained by solving the nonlinear optimization problem [36]:

(16) $$\begin{array}{cc} \min f(G) & ={∥Z^{\mathrm{ideal}}-W^{T}G∥}^{2},\\ s.t.\; & 0\leq G_{l}\leq m.E_{g},\;\forall l=1,2,\ldots ,L,\\ \; & \sum_{l=1}^{L}G_{l}=E_{s}. \end{array}$$

where W is a $3l\times N_{int}$ columnwise circulant matrix with first column defined as ${\left[1_{L\times 1}^{T},0_{2L\times 1}^{T}\right]}^{T}$ and $G={\left[G_{1},G_{2},\ldots ,G_{L},0_{L\times 1}^{T},G_{1},G_{2},\ldots ,G_{L}\right]}^{T}$. Here, $E_{g}$ represents the energy constraint for each pulse $g_{i}\left(t\right)$, satisfying $E_{g_{i}}\leq E_{g}\leq E_{s}$. The optimization is solved using sequential quadratic programming, where the Hessian of the Lagrangian is updated at each iteration using the BFGS formula [58,59]. The relationship $W^{T}G$ can be expressed as [36]

(17) $$W^{T}G=Z,$$

with

(18) $$\begin{array}{cc} G_{l} & =\int_{t_{s}+\frac{\left(l-1\right)T_{b}}{N_{int}}}^{t_{s}+\frac{lT_{b}}{N_{int}}}x^{2}\left(t\right)\;dt=\sum_{i=(l-1)m+1}^{lm}\int_{T_{p}}g_{i}^{2}\left(t\right)\;dt\\ \; & =\sum_{i=(l-1)m+1}^{lm}E_{g_{i}}. \end{array}$$

The optimal p ulse energies can take continuous values. While the synchronization starting point can technically fall anywhere within $\left[0,T_{b}/2\right]$, it is sufficient to select it within $\left[0,T_{b}/2N_{int}\right]$ without loss of generality.

## 5. Simulations

### 5.1. Optimal Preamble Energy Allocation

**Figure 6:**
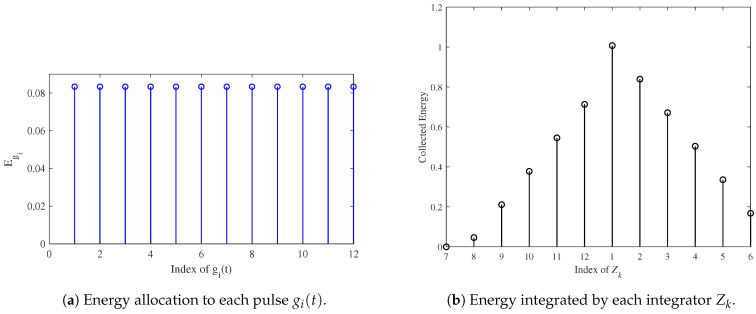
Preamble energy allocation and integrator output [36].

The basic pulse, p(t), is modeled as a $2.5\times 10^{3}\;\mathrm{fs}$ long first derivative of a Gaussian pulse. Figure 6a illustrates the energy allocated to each $g_{i}\left(t\right)$, determined by solving a constrained nonlinear optimization problem. Figure 6b shows the energy collected by the integrators. As designed, the first integrator collects the maximum energy, making its corresponding time the optimal ToA.

**Figure 7:**
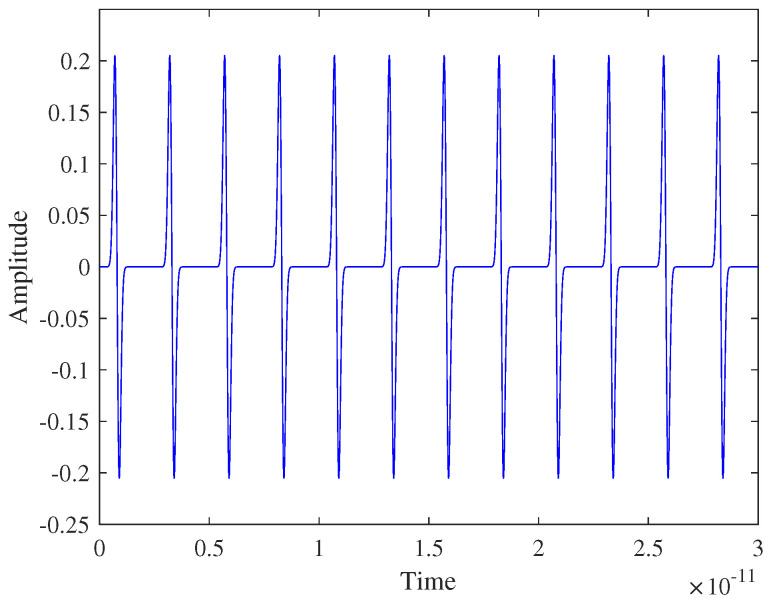
Designed p reamble signal.

Figure 7 depicts the preamble signal, representing only the signaling component of the complete preamble. Each pulse in the preamble is assigned equal energy, ensuring effective synchronization. The preamble signal consisting of multiple equal-amplitude pulses, as derived from (14), is transmitted before data communication to facilitate synchronization by allowing the receiver to estimate the ToA. Figure 6 illustrates the design rationale and validation for the preamble signal, as shown in Figure 7. The energy allocation (Figure 6a) and energy collection behavior (Figure 6b) ensure that the preamble signal in Figure 7 effectively supports synchronization by maximizing energy collection in the integrators.

### 5.2. Synchronization Performance

To evaluate the performance of the proposed scheme, we utilize the synchronization probability, $P_{syn}$, and the average normalized mean squared error, 〈NMSE〉. The 〈NMSE〉 quantifies the synchronization accuracy by measuring the mean squared error between the estimated ToA and the ideal ToA. Mathematically, it is defined as [40]

(19) $$\langle \mathrm{NMSE}\rangle =E\left[{\left(\frac{|{\hat{t}}_{syn}-t_{syn}|}{T_{b}}\right)}^{2}\right],$$

where E(·) is the expectation operator. The THz-WiNoC channel model described in Section 3 is employed, considering a CMOS-based chip with varying LoS distances between the horizontal dipole and the observation point. A high-resistivity silicon substrate of 5 kΩ·cm is used [28]. For the simulations, the number of integrators, $N_{int}$, is fixed at twelve, and the total number of preamble repetitions is set at ten. A total of $10^{4}$ Monte Carlo runs are conducted, with other simulation parameters listed in Table 1.

**Figure 8:**
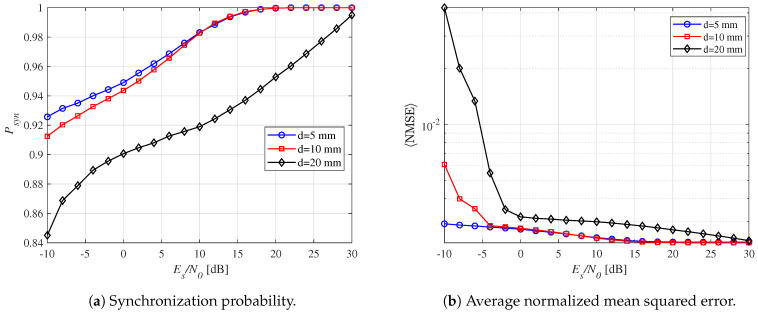
Synchronization perf ormance according to the distance between the dipole and observation point for a fixed $N_{int}=12$ and N=10.

Figure 8a shows the synchronization probability for three distances in the THz-WiNoC channel. As $E_{s}/N_{0}$ increases, synchronization probability also improves. For a distance of up to 10 mm, synchronization probability exceeds 0.9 across the signal-to-noise ratio (SNR) regime. At a distance of 20 mm, the synchronization probability slightly decreases due to greater path loss, requiring a higher SNR to maintain performance. Figure 8b presents the 〈NMSE〉 for the same distances, incorporating both $P_{syn}$ and ${\hat{t}}_{syn}$ results. The ${\hat{t}}_{syn}$ values are confined within the error range ${\hat{t}}_{syn}\in \left[t_{syn}-2500\;\mathrm{fs},t_{syn}+2500\;\mathrm{fs}\right]$ across all distances. Overall, the three curves exhibit a decreasing trend in error as SNR increases. At *d* = 0.5 mm, the synchronization error is the lowest, reflecting optimal synchronization performance at shorter distances. Conversely, at *d* = 20 mm, synchronization performance deteriorates, resulting in the highest error.

**Figure 9:**
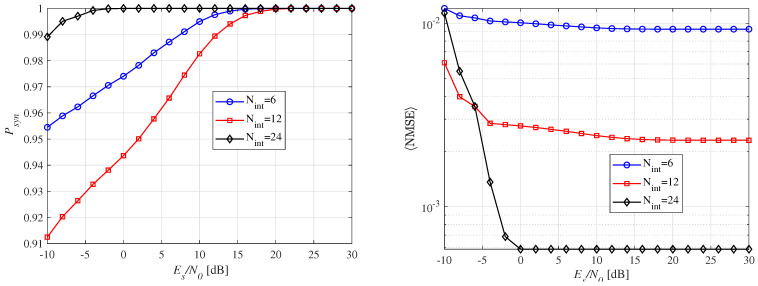
Synchronization performance according to the number of integrators for a fixed distance of d= 10 mm and N=10.

Figure 9 illustrates the $P_{syn}$ and 〈NMSE〉 with respect to the number of integrators. In Figure 9a, a nonlinear relationship between $P_{syn}$ and $N_{int}$ is observed. Specifically, a small number of integrators, such as $N_{int}$ = 6, achieves better performance in the low-SNR region with a probability greater than 0.95. This can be attributed to the fact that a small number of integrators leads to wider sub-windows, improving energy collection and, consequently, performance. However, it is important to note that a smaller $N_{int}$ results in a higher 〈NMSE〉, as the wider sub-windows increase the synchronization error, affecting the accuracy of the estimated ToA (${\hat{t}}_{syn}$). For $N_{int}=24$, optimal performance is observed across the entire SNR range. This is because the width of the sub-window becomes equal to the preamble pulse, meaning that only the signal energy is squared and integrated, providing a clearer distinction between the noise and signal. As a result, very little noise is captured, even in the low-SNR region. As shown in Figure 9b, increasing the number of integrators improves 〈NMSE〉. This improvement is due to the enhanced resolution of the sub-windows, stemming from their shorter widths, leading to higher synchronization performance. This behavior demonstrates the trade-off between the number of integrators, $P_{syn}$, and SNR, which influences the optimal choice of integrators for a given THz-WiNoC system. In practice, coarse synchronization can be achieved with fewer integrators, resulting in favorable $P_{syn}$, and then fine synchronization can be performed within the candidate region using more integrators with narrower sub-windows for higher precision. This approach proves more effective for practical synchronization operations.

**Figure 10:**
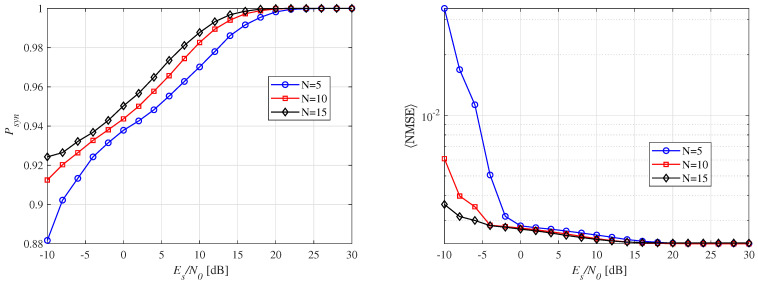
Synchronization performance according to the number of preamble repetitions for a fixed distance of d= 10 mm and $N_{int}=12$.

Figure 10a presents synchronization performance for varying numbers of preamble repetitions, *N*. As *N* increases, synchronization probability improves, particularly in low-SNR regions. For example, with N=10, a synchronization probability of over 0.9 is achieved in low-SNR regions, and over 0.98 in high-SNR regions (10 dB or more). For high-reliability THz-WiNoC communications, perfect synchronization is essential, and the results are promising in this context. The observed synchronization trend can be attributed to the fact that repeating the preamble signal multiple times increases the amount of signal energy captured by the sub-windows, even in low signal power conditions. Thus, increasing the value of *N* enables the receiver to better combat noise and achieve improved synchronization. Figure 10b shows the average NMSE, which, like $P_{syn}$, depends on *N*. Overall, a higher number of preamble repetitions leads to more reliable synchronization due to enhanced detection accuracy and noise averaging. However, it may result in a synchronization delay.

### 5.3. Challenges in Hardware Feasibility

Implementing the proposed algorithm on a real-world hardware platform presents several challenges. While significant progress has been made in THz nanocommunications over the past decade, the field remains in its early stages. For instance, practical THz transceivers still face limitations related to power efficiency and signal sensitivity. Developing compact, energy-efficient nanotransmitters and nanoreceivers for the THz band remains a key challenge [6,60,61]. In pulsed THz systems, components such as analog front ends, plasmonic nanoantennas, and analog-to-digital/digital-to-analog converters must operate at femtosecond resolutions, adding considerable complexity. The proposed synchronization scheme relies on multiple energy-collecting integrators to determine the ToA, which requires high-speed analog circuits capable of performing integration on the femtosecond time scales. Currently, this is both technologically demanding and resource-intensive. Furthermore, integrating high-speed electronics and plasmonics elements into nanocommunication systems introduces thermal challenges. As a result, efficient thermal management and low power consumption are essential, yet remain unresolved issues in THz hardware. As THz nanocommunication technology advances, future research will likely focus on implementing prototypes on platforms such as FPGAs or ASICs. This will enable the validation of the proposed scheme under real-world conditions and help identify any practical limitations that may arise.

## 6. Conclusions

This study presents a preamble-aided synchronization scheme for ToA estimation in THz-WiNoC communication systems. The proposed scheme is based on a straightforward, noncoherent energy-detection receiver. A CMOS chip is considered with a flip-chip architecture. The THz-WiNoC channel model is based on the EM field analysis in the THz band. At the receiver, energy from the preamble signal is collected across several integrators. The integrator that accumulates the most energy is used to determine the ToA of the symbol. A mathematical model of the proposed scheme is introduced, and its performance is evaluated in terms of synchronization probability and average NMSE. Simulation results highlight the significance of the distance in WiNoC communications. Additionally, performance can be enhanced by adjusting the number of integrators and preamble repetitions. This work lays the foundation for further research into modulation and channel access mechanisms for THz-band WiNoC communications.

## Figures and Tables

**Table 1:** Default s imulation parameters.

Parameter	Values
Distance (*d*)	10 mm
Pulse width ($T_{g}$)	2500 fs
Pulse variance (σ)	100 fs
Pulses in one preamble, $N_{s}$	12
Number of integrators, $N_{int}$	12
Number of repetitions of the basic preamble, *N*	10

## Data Availability

The original contributions presented in the study are included in the article. Further inquiries can be directed to the corresponding author.
